# Characterization of the Initial Fouling Layer on the Membrane Surface in a Membrane Bioreactor: Effects of Permeation Drag

**DOI:** 10.3390/membranes9090121

**Published:** 2019-09-17

**Authors:** Shengli Wang, Xin Lu, Lanhe Zhang, Jingbo Guo, Haifeng Zhang

**Affiliations:** 1 School of Chemistry Engineering, Northeast Electric Power University, Jilin 132012, China; taijixiaomao@163.com (S.W.); 13944279096@163.com (X.L.); zhangbuleriver@163.com (L.Z.); 2 School of Civil and Architecture Engineering, Northeast Electric Power University, Jilin 132012, China; gjbeepu@163.com

**Keywords:** membrane bioreactors, extracellular polymeric substances, initial fouling layer, permeation drag, interaction energy

## Abstract

In this study, the properties of the initial fouling layer on the membrane surface of a bioreactor were investigated under different operating modes (with or without permeate flux) to improve the understanding of the effect of permeation drag on the formation of the initial fouling layer. It was found that protein was the major component in the two types of initial fouling layers, and that the permeation drag enhanced the tryptophan protein-like substances. The attraction of the initial foulants to the polyvinylidene fluoride (PVDF) membrane was ascribed to the high zeta potential and electron donor component (*γ−*) of the membrane. Thermodynamic analyses showed that the permeation drag-induced fouling layer possessed high hydrophobicity and low *γ−*. Due to permeation drag, a portion of the foulants overcame an energy barrier before they contacted the membrane surface, which itself possessed a higher fouling propensity. A declining trend of the cohesive strength among the foulants was found with the increasing development of both fouling layers.

## 1. Introduction

Membrane bioreactors (MBRs) are gaining worldwide attention as a promising solution for wastewater treatment. These reactors have many advantages over conventional activated sludge systems, such as a smaller footprint and better effluent quality, which enables reuse [1,2]. However, membrane fouling, especially biofouling, has been the main obstacle for the wide application of MBRs [3]. It is widely known that biofouling starts with the deposition of foulants during the initial stages of membrane filtration, resulting in the development of additional fouling layers on the membrane surface [4]. The initial fouling layer affects the intrinsic properties of the membrane and thereby the subsequent development of additional layers [5]. Moreover, it is noteworthy that the flux decay in the early stage of filtration accounts for a significant portion of the permeate flux (approximately 30–50%) under constant pressure [6]. Thus, the initial fouling layer plays a significant role in the membrane filtration process and requires more consideration.

Compared with sludge flocs, extracellular polymeric substances (EPS) adhere relatively easily to the membrane surface, and their flexible characteristics result in readjustment to a lower energy state during the attachment process [7]. These changes in surface energy are often the precursors for sludge floc attachment. The EPS have a complex composition and consist of proteins, polysaccharides, extracellular deoxyribonucleic acids (eDNA), humic acid, etc. [8]. Although researchers have found a remarkable correlation between the EPS components and membrane fouling in MBRs [2,3,5,8], a fundamental understanding of the particular EPS components responsible for initial biofouling is still lacking.

It has been recently reported that the adhesion of foulants to the membrane surface in MBRs is a thermodynamic process that can be described by the extended Derjaguin-Landau-Verwey-Overbeek (XDLVO) theory [9,10,11,12] based on the sum of the London-van der Waals (LW), electrostatic double layer (EL), and acid-base (AB) interaction energies. The attachment of foulants to the membrane surface largely depends on the interaction forces between the foulant surface and membrane surface [13]. In disturbed systems such as MBRs, there are two opposite forces that control the motion of foulants towards the membrane surface: permeation drag, which is generated by permeate flux and back transport, and consist of Brownian diffusion, inertial lift, and shear-induced diffusion [14,15,16]. For a given aeration condition, the permeate flux is expected to significantly affect the adhesion process from a hydrodynamic viewpoint and then plays a dominant role in the formation of the initial fouling layer. However, information on the effect of permeation drag on the properties of the initial fouling layer in an MBR is scarce, and this topic deserves further study.

The objective of this study was to investigate the effects of the permeation drag on the characteristics of the initial fouling layer. Two typical fouling layers were developed under normal flux (10 L/m^2^·h) and no flux (0 L/m^2^·h) conditions for 24 h in an MBR operating under stable conditions. The initial foulants were collected from the membrane surface and the EPS compositions were characterized in terms of the protein, polysaccharide, and eDNA contents. The interfacial interactions of the membrane and sludge flocs with the initial foulants and the internal interactions of the foulants and sludge flocs were assessed using XDLVO models. The results of this study are expected to provide a better understanding of the initial fouling layer formation in the MBR.

## 2. Materials and Methods

### 2.1. Experimental Setup and Operation

**Figure 1:**
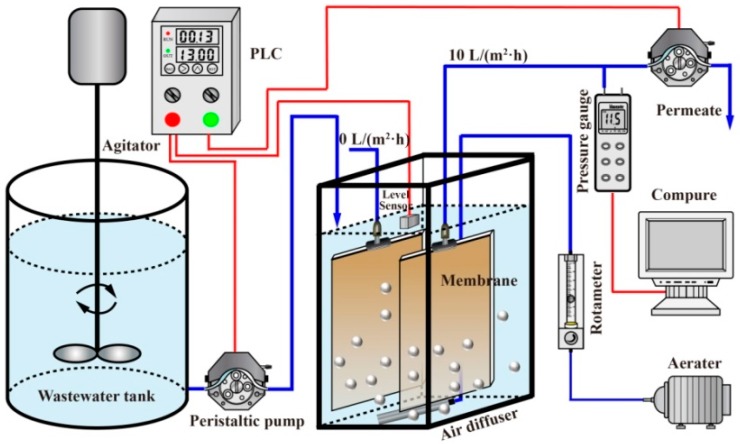
Schematic diagram of the submerged MBR experimental setup.

A lab-scale 7-L MBR was operated at room temperature of 24 ± 2 °C (Figure 1). The MBR was equipped with two submerged flat-sheet membrane modules, which were made of polyvinylidene fluoride (PVDF) (Shanghai SINAP Co. Ltd., Shanghai, China) with a nominal pore size of 0.1 μm and a total filtration area of 0.2 m^2^ (0.1 m^2^ each). PVDF membranes were selected because they are representative membrane materials and have been widely used in MBR applications [17]. 

The MBR was fed with synthetic municipal wastewater (glucose 280 mg/L; NH_4_Cl 100 mg/L; KH_2_PO_4_ 20 mg/L; NaHCO_3_ 172 mg/L; CaCl_2_ 10 mg/L; MgSO_4_·7H_2_O 50 mg/L; FeCl_3_ 0.375 mg/L; CuSO_4_·4H_2_O 0.1 mg/L; NaMoO_4_·2H_2_O 0.15 mg/L; MnSO_4_·H_2_O 0.13 mg/L; ZnCl_2_ 0.23 mg/L; CoCl_2_·6H_2_O 0.42 mg/L). The details of the formulation were described in our previous study [2]. The average concentrations of the chemical oxygen demand (COD), ammonia nitrogen (NH_4_^+^–N), and total phosphorus (TP) of the synthetic wastewater were 212.18 ± 2.51 mg/L, 19.10 ± 2.90 mg/L and 5.72 ± 0.62 mg/L (Table 1). The membrane module was operated with a permeate flux of 10 L/m^2^·h and the permeate flowing through the membrane module was continuously withdrawn using a peristaltic pump (Model BT-100, Baoding Longer Precision Pump Co., Ltd., Baoding, China) running in a 13-min-on and 2-min-off mode. Aeration was provided continuously underneath the membrane module at 5 L/min to supply a hydraulic shear force and oxygen for the biomass; the specific air demand per membrane surface (SAD_m_) was 1.5 m^3^/m^2^·h. The hydraulic retention time (HRT) and sludge residence time (SRT) were maintained at 8 h and 30 d, respectively.

Prior to the experiment, the first membrane modules were firstly immersed in ultrapure water for 48 h to obtain virgin membranes. Then, the membrane module was inserted into the MBR tank, which was operated with normal flux (10 L/m^2^·h) in an intermittent mode (13-min-on and 2-min-off). In parallel, the second identical module was also inserted into the MBR tank without permeate production (0 L/m^2^·h). Two membrane modules were simultaneously removed from the tank and replaced with new membrane modules after 24 h. The experimental process was repeated six times.

### 2.2. Contact Angle and Zeta Potential Analysis

The contact angles of the activated sludge sample, the virgin and fouled membranes were measured using the method described by Hong et al. [18]. In brief, the virgin and fouled membranes were first cut into small pieces (2 cm × 2 cm), which were mounted on a slide. The activated sludge sample was collected from the MBR tank; the sample was first filtered through a membrane with 0.45 μm pore size, and the deposit was pressed between two slides to form a flat surface. Thereafter, the samples were dried in a desiccator for 24 h to remove surplus water. Three probe liquids including ultrapure water, diiodomethane, and glycerol were used for the contact angle measurements. The static contact angles of the probe liquids on the prepared samples were measured using a contact angle meter (JC2000D1, Shanghai Powereach Co., Ltd., Shanghai, China) and the sessile drop method.

The zeta potentials of the foulant and sludge flocs samples were measured by with a Zetasizer (JS94H, Shanghai Powereach Co., Ltd., Shanghai, China) and using the electrophoretic mobility method. A Zeta 90 Plus Zeta Potential Analyzer (Brookhaven Instruments, Austin, TX, USA) was used to analyze the zeta potential of the membrane surface.

### 2.3. EPS Extraction and Analysis

The EPS extraction protocol in this study was a modification of a protocol used in a previous study [19]. In brief, a 25 mL sludge sample was centrifuged at 4000 rpm for 5 min at 4 °C and the supernatant was collected as S-EPS. The residual sludge in the centrifuge tube was resuspended to its original volume of 25 mL with the NaCl solution (0.9% NaCl). Immediately, the sludge suspension was mixed by a vortex mixed (G-560, Scientific Industries, Inc., Bohemia, New York, NY, USA) for 1 min and then centrifuged at 4000 rpm for 10 min at 4 °C. The supernatant was collected as LB-EPS. The residual sludge pellet in the centrifuge tube was re-suspended to its original volume of 25 mL with the NaCl solution and then subjected to a water bath at 80 °C for 30 min. Finally, it was centrifuged at 12,000 rpm for 20 min to collect TB-EPS. For the foulant samples, the total EPS were extracted using the method described by Zhang et al [20]. The foulant samples were scraped from the membrane surface with a blade, then re-suspended with the NaCl solution. The mixed liquor was then subjected to heat treatment (100 °C, 1 h) and centrifuged at 6000 rpm for 30 min. The centrifuged supernatant was regarded as the total EPS solution.

The protein content was determined using the Coomassie brilliant blue method [21] with bovine serum albumin (BSA) as the standard and the polysaccharide content was determined using the anthrone method [22] with glucose as a standard. The eDNA content was obtained using the diphenylamine colorimetric method with calf thymus DNA as the standard [23]. Fluorescence excitation-emission matrix (EEM) measurements of the EPS solution samples were performed using a luminescence spectrometer (RF-6000, Shimadzu Co., Ltd., Tokyo, Japan); the excitation (Ex) ranged from 200 to 400 nm at 5 nm sampling intervals and the emission (Em) ranged from 200 to 500 nm at 5-nm sampling intervals. The Ex and Em slits were set at 3-nm band pass.

### 2.4. Surface Thermodynamics and XDLVO Approach

The surface tension parameters ($\gamma^{LW}$, $\gamma^{+}$ and $\gamma^{-}$) of the virgin membrane, fouled membrane, initial foulants, and sludge flocs were calculated by solving a set of three Young’s equations [24]. According to the value of $\gamma^{LW}$, $\gamma^{+}$, $\gamma^{-}$, $\gamma^{AB}$ and $\gamma^{Tot}$ were calculated. The $\Delta G_{adh}$ is related to the adhesion of the initial foulants on the membrane surface, while $\Delta G_{coh}$ is the cohesion energy per unit area between the foulants and sludge flocs. The more negative the values of $\Delta G_{adh}$ and $\Delta G_{coh}$, the stronger the adhesion and cohesion are [9]. The surface hydrophobicity/hydrophilicity ($\Delta G_{sws}$) was evaluated using the free energy of interaction between two identical surfaces immersed in water. If $\Delta G_{sws}$< 0, the surface is considered hydrophobic, and vice versa [9]. In XDLVO theory, the total energy of the interaction ($U_{\mathrm{Tot}}$) is the summation of the EL energy ($U_{EL}$), the LW interaction energy ($U_{LW}$) and the AB interaction ($U_{AB}$) [13]. The physicochemical interactions energies ($U_{EL}$, $U_{LW}$ and $U_{AB}$) were expressed as a function of the separation distance *d* and were calculated according to Zhang et al. [25]. The detailed calculation process is described in our Supporting Information.

### 2.5. Others

Standard analytic methods were used to measure the COD, NH_4_^+^–N, TP, mixed liquor suspended solids (MLSS), and mixed liquor volatile suspended solids (MLVSS) [26]. 

## 3. Results and Discussion

### 3.1. Composition and Content of EPS Fractions in the Initial Fouling Layer

The lab-scale MBR system was continuously operated for more than 100 d. The average removal efficiency of the COD and NH_4_^+^–N during the stable operation period were 92.84% and 92.04%, respectively (Table 1). The EPS contents were quite stable during this period and the total S-EPS, LB-EPS, and TB-EPS were 5.16 ± 1.21 mg/g SS, 5.60 ± 1.92 mg/g SS and 41.56 ± 0.65 mg/g SS, respectively (Table 2).

**Figure 2:**
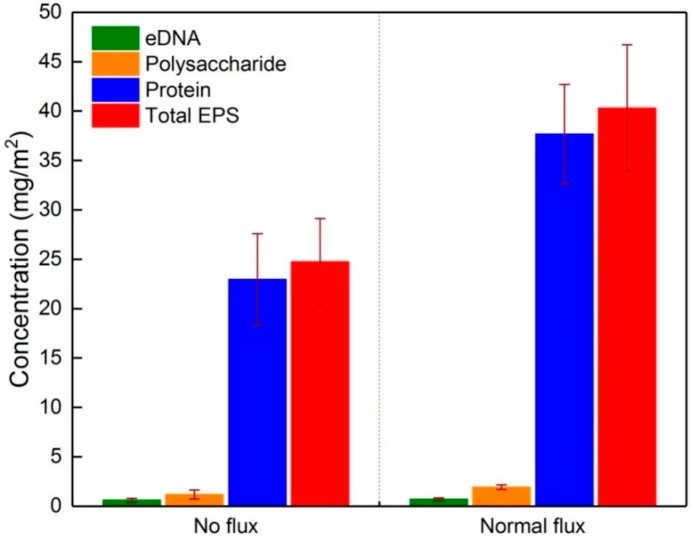
EPS fractions in initial fouling layers under no flux (0 L/m^2^·h) and normal flux (10 L/m^2^·h) conditions.

Figure 2 shows the concentrations of EPS fractions in the two types of initial fouling layers. It was observed that both of the initial fouling layers contained similar EPS fractions, whereas the total EPS amount was 62.8% higher under normal flux (with permeation drag) than no flux conditions, e.g., the polysaccharide, eDNA, and protein concentrations were 1.31, 0.89, and 24.36 mg/m^2^ under no flux conditions but were 12, 20, and 63.6% higher under normal flux conditions, respectively. The protein was more abundant than the other components under both flux conditions, accounting for about 92.8% (no flux) and 93.5% (normal flux) of total EPS contents on the membrane surface. Matar et al. also found that the fouling layer on the membrane surface was mainly composed of protein after 24 h operation [5], which agreed with our findings following same short membrane operation time.

Fluorescence EEM spectroscopy was used to characterize the samples (Figure S1); the peak intensities are listed in Table 3. Two distinct peaks were observed in both initial foulants; the high-intensity peak A related to tryptophan protein-like substances was detected at Ex/Em of 280/345 nm and the low-intensity peak B related to aromatic protein-like substances was detected at Ex/Em of 235/340 nm [27]. This suggested that the protein-like substances indeed facilitated the formation of the initial layer on the membrane surface. The intensity of peak A was 79.2% higher under normal flux conditions and was significantly higher than the intensity of peak B (34.7% higher), indicating that the presence of the permeation drag force had a more important influence on the adhesion of tryptophan protein-like substances than that of other protein fractions on the membrane surface. Similar results also were observed by Wang et al. [28], who found that tryptophan proteins were more likely to accumulate on the membrane surface under permeate flux conditions, highly suggesting the significant role of permeation drag in the buildup of tryptophan protein-like substances on the membrane surface.

### 3.2. Surficial Properties of Membrane and Initial Fouling Layer

The initial foulant adhesion on the virgin membrane surface resulted from the surficial properties of the foulants and membrane [29]. The surficial properties (i.e., the contact angle and zeta potential) were significantly different for the fouled membranes and virgin membrane (Table 4), indicating that the foulants had completely covered the membrane surface and altered the surface characteristics of the virgin membrane, even under no flux conditions. All four materials were negatively charged, and the absolute values of the zeta potential followed the order virgin membrane > sludge flocs > fouled membrane (normal flux) > fouled membrane (no flux). The virgin membrane surface possessed a more negative charge (−31.22 mV on average) than the other materials, thereby increasing the electrostatic repulsion of high negatively charged foulants. Therefore, fewer foulants with a high negative change adhered to the membrane surface, resulting in low negatively charged fouling layer. The zeta potential of the fouled membrane surface under no flux conditions (−9.59 mV) was significantly lower than that of the fouled membrane under normal flux conditions (−15.59 mV), indicating that the permeation drag promoted the adhesion of high negatively charged foulants on the membrane surface.

The surface thermodynamic parameters of the four materials were calculated (Table 5) according to the data presented in Table 4. The virgin membrane exhibited a relatively high electron donor component ($\gamma^{-}$), indicating that the PVDF membrane showed a Lewis base character because fluorine atoms in the membrane contain isolated electron pairs [13], thus inducing potential interaction with attractive Lewis acids. With respect to the EPS fractions, the most typical acids are a variety of amino acids contained in proteins. This is the main reason for proteins accumulating in both fouling layers (Figure 2). Additionally, eDNA is known to be a naturally acidic substance and also has a high potential to attach to the PVDF membrane surface. However, a low eDNA level was found in both fouling layers in this study; for example, the eDNA content averaged 0.68 mg/m^2^ and 0.72 mg/m^2^ in the initial fouling layers under no and normal flux conditions, respectively (Figure 2). This might be interpreted in terms of the high negative charge of eDNA [30] that suppressed its adherence to the PVDF membrane surface. In conjunction with the results presented in Section 3.1, this indicates that the composition of the initial foulants was mainly associated with the membrane properties and was independent of the permeation drag.

Recent studies have indicated that the $\gamma^{-}$ of the membrane surface is an effective indicator to predict adsorptive fouling in MBRs; a high $\gamma^{-}$ can confer high surface anti-adhesion ability [11,12]. It was evident that the surface $\gamma^{-}$ of the two types of fouled membranes were lower than that of the virgin membrane, which indicated that the formation of the initial fouling layer enabled subsequent cohesive fouling. Compared to the fouled membrane without permeation drag, a 41.5% reduction in the surface $\gamma^{-}$ was observed for the fouled membrane under normal flux conditions, suggesting that the permeation drag-induced fouling layer had a higher fouling potential. All four materials were hydrophobic based on the negative values of $\Delta G_{sws}$. Compared with the fouled membrane under no flux conditions (−34.77 mJ/m^2^), the presence of the permeation drag force resulted in greater hydrophobic foulant accumulation on the membrane surface (−44.34 mJ/m^2^). Although the two types of foulants had similar compositions in EPS fractions, they exhibited different surface properties, which was likely attributable to their contents, the zeta potential, $\gamma^{-}$, and the hydrophobicity; these differences highly suggested the important role of permeation drag during the formation of the initial fouling layer.

### 3.3. Changes in the Interaction Energy during Membrane Fouling 

**Figure 3:**
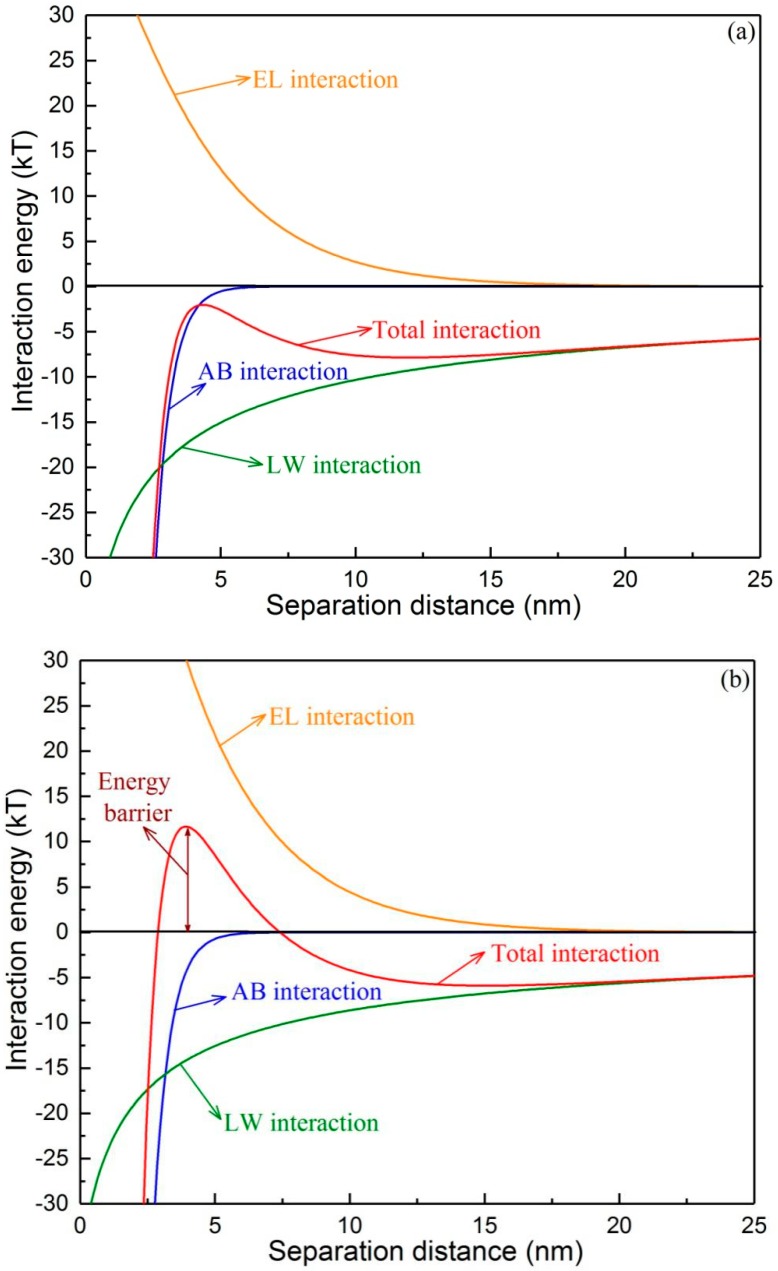
Profiles of interaction energies between membrane and initial foulants: (**a**) under no flux condition (0 L/m^2^·h) and (**b**) normal flux condition (10 L/m^2^·h).

The different interfacial energy components versus the separation distance between the two types of initial foulants and the virgin membrane are shown in Figure 3. It was evident that for the two types of foulants, the AB and LW interaction energies were attractive and played important roles at a short separation distance (<6 nm), whereas the EL interaction energy was repulsive due to the negatively charged membrane and initial foulants (Table 4). Under no flux conditions (Figure 3a), the total interaction energy was continuously attractive and covered the whole separation distance without any barrier, indicating that the foulants would spontaneously approach the membrane and actively adhere to the membrane surface. It was observed that a repulsive energy barrier existed for the permeation drag-induced foulants (Figure 3b), which suggested that these foulants encountered this energy barrier before they had the opportunity to adhere to the membrane surface. It is reasonable to believe that the permeation drag force helped the foulants to overcome this barrier to reach the membrane surface.

**Figure 4:**
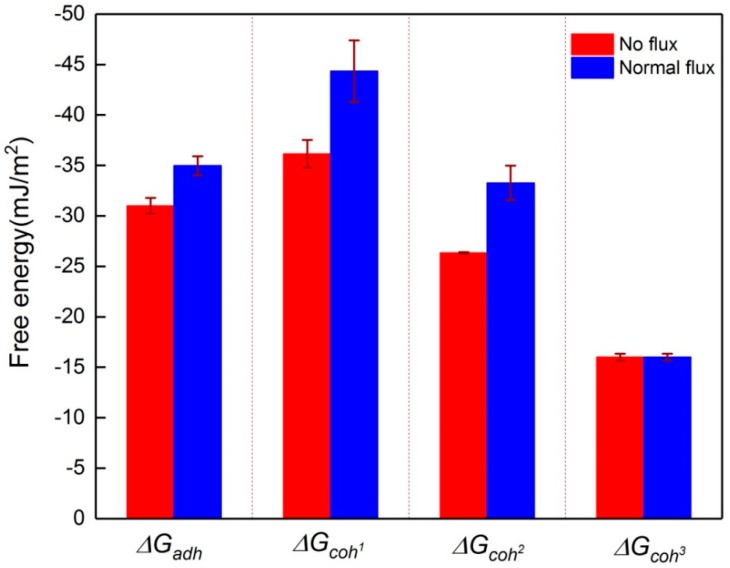
Comparisons of interaction energy of adhesion ($\Delta G_{adh}$) of virgin membrane- initial foulants, cohesion energy of initial foulants-initial foulants ($\Delta G_{{coh}^{1}}$), initial foulants-sludge flocs ($\Delta G_{{coh}^{2}}$) and sludge flocs-sludge flocs ($\Delta G_{{coh}^{3}}$) under no flux (0 L/m^2^·h) and normal flux (10 L/m^2^·h) conditions.

The interaction energy between the foulants and membrane is divided into two types: one is the attachment strength between the initial foulants and the virgin membrane (adhesion) and the other is the cohesive strength between the foulants themselves (cohesion) [10,31,32]. Considering the significant differences between the initial foulants and sludge flocs, the cohesion energy of the initial foulants-initial foulants ($\Delta G_{{coh}^{1}}$), initial foulants-sludge flocs ($\Delta G_{{coh}^{2}}$) and sludge flocs-sludge flocs ($\Delta G_{{coh}^{3}}$) were predicted in this study. As shown in Figure 4, both initial foulants exhibited a high adhesion to the membrane, i.e., −34.9 mJ/m^2^ and −31.0 mJ/m^2^ under normal and no flux conditions, respectively. They also exhibited high self-cohesive strength (−44.3 mJ/m^2^ for normal flux and −36.1 mJ/m^2^ for no flux, respectively). These results implied that it was difficult to detach the initial fouling layer from the membrane surface by shearing due to the high interaction energy, which confirmed that back-flushing was more effective than cross-flow for the removal of the initial foulants [16]. It was interesting that a decreasing trend of the cohesive strength was observed with the membrane fouling development under both flux conditions; for example, the cohesive interaction energies between the foulants decreased in the following order: $\Delta G_{{coh}^{1}}$ > $\Delta G_{{coh}^{2}}$> $\Delta G_{{coh}^{3}}$ for both types of foulants, implying that the removal of the outer foulants seems to be easier due to the lower cohesive energy among them [33].

## 4. Conclusions

The properties of the initial fouling layer on the membrane surface with and without permeate flux conditions were investigated and compared in this study. It was found that the content of the initial foulants were determined by the permeation drag force. Protein was the major EPS fraction in both types of fouling layers and the permeation drag force resulted in higher tryptophan protein content in the EPS fractions. The attraction of the initial foulants to the membrane was ascribed to the high zeta potential and surface $\gamma^{-}$ component of the membrane. Thermodynamic analyses showed that a fraction of the foulants overcame a positive energy barrier in the presence of the permeation drag force before they contacted the membrane surface, which possessed high hydrophobicity, high negative charge, and low $\gamma^{-}$. Both initial foulants exhibited strong adhesion to the membrane and the cohesive strength gradually decreased with further fouling development. The results of this study shed light on the roles of the membrane and permeation drag force during the formation of the initial fouling layer. Additional studies are required to exploit the combined strategy of initial fouling mitigation as a result of a reduction in tryptophan protein content in the bulk sludge and a membrane surface with high zeta potential.

## Figures and Tables

**Table 1:** A summary of the MBR performance ^a^.

Parameter	Content
COD in influent (mg/L)	212.18 ± 2.15
NH4+–N in influent (mg/L)	19.10 ± 2.90
TP in influent (mg/L)	5.72 ± 0.62
COD removal (%)	92.84 ± 4.31
NH4+–N removal (%)	92.04 ± 6.04
TP removal (%)	19.19 ± 1.09
MLSS (mg/L)	4.50 ± 0.27
MLVSS (mg/L)	3.46 ± 0.13
MLVSS/MLSS(%)	0.77 ± 0.01

^a^ Each value represented the average of all measurements (*n* = 10) and ± was absolute deviation from the average. Mixed liquor suspended solids: MLSS, mixed liquor volatile suspended solids: MLVSS

**Table 2:** The distribution of EPS fractions during the experimental period ^a^.

Components	EPS Fractions	Content (mg/g SS)
eDNA	S-EPS	0.65 ± 0.21
	LB-EPS	0.64 ± 0.28
	TB-EPS	2.13 ± 0.54
Protein	S-EPS	2.27 ± 1.58
	LB-EPS	3.12 ± 0.46
	TB-EPS	18.07 ± 2.23
Polysaccharide	S-EPS	2.24 ± 0.29
	LB-EPS	1.84 ± 0.44
	TB-EPS	21.36 ± 1.16

^a^ Each value represented the average of all measurements (*n* = 10) and ± was absolute deviation from the average.

**Table 3:** The peaks locations and intensities of the fluorescence EEM spectra for initial foulants on membrane surface with no flux (0 L/m^2^·h) and normal flux (10 L/m^2^·h).

Samples	Peak A		Peak B	
	Ex/Em (nm)	Intensity (a.u.)	Ex/Em (nm)	Intensity (a.u.)
No flux	280/345	108.80	235/340	23.86
Normal flux	280/345	195.02	235/340	32.15

**Table 4:** Zeta potential and contact angle of three probe liquids data for virgin membrane, fouled membranes, and sludge flocs ^a^.

Materials	Contact Angle (°)			Zeta Potential (mV) ^b^
	Water	Glycerol	Diiodomethane	
Virgin membrane	58.86 ± 2.27	53.08 ± 1.17	20.69 ± 0.99	−31.22 ± 0.13
Fouled membrane (no flux)	72.51 ± 0.29	69.45 ± 1.24	38.79 ± 3.34	−9.59 ± 1.68
Fouled membrane (normal flux)	78.55 ± 4.39	70.32 ± 5.54	45.43 ± 0.99	−15.59 ± 4.95
Sludge flocs	78.32 ± 2.13	85.52 ± 1.39	57.59 ± 3.45	−26.23 ± 1.73

^a^ Each value represented the average of all measurements (*n* = 6) and ± was absolute deviation from the average. ^b^ Zeta potential measured using 10 mM NaCl as ionic solution, and presented for pH 7.0.

**Table 5:** Surface tension parameters and cohesion energy (mJ/m^2^) for virgin membrane, fouled membranes and sludge flocs ^a^.

Materials	$\gamma^{LW}$	$\gamma^{+}$	$\gamma^{-}$	$\gamma^{AB}$	$\gamma^{Tot}$	$\Delta G_{sws}$ (mJ/m^2^)
Virgin membrane	45.86	0.13	17.45	2.98	48.84	−25.22
Fouled membrane (no flux)	40.21	0.02	13.26	−0.95	39.26	−34.77
Fouled membrane (normal flux)	36.78	0.06	7.76	1.37	38.15	−47.39
Sludge flocs	29.96	0.67	19.57	−7.24	22.72	−15.98

^a^ Each value represents the average of all calculations (*n* = 6).

## Supplementary Materials

The following are available online at https://www.mdpi.com/2077-0375/9/9/121/s1, Figure S1: EEM fluoreacence spectra of EPS extracted from the initial fouling layer on membrane with no flux (0 L/m^2^·h) and normal flux (10 L/m^2^·h).

## Nomenclature

$\gamma^{-}$	surface tension parameter
ΔG	interaction energy per unit area
U	interaction energy
*AB*	Lewis acid-base
*EL*	electrostatic double layer
*LW*	Lishitz-van der Waals
Superscripts	
*tol*	total
+	electron acceptor
−	electron donor
Subscripts	
*adh*	adhesion between foulants and membrane
*coh*	cohesion among foulants
*s*	solid
*w*	water
