# Supplementary material for: Characterization of the Initial Fouling Layer on the Membrane Surface in a Membrane Bioreactor: Effects of Permeation Drag
Source: Membranes (Basel). 2019 Sep 17;9(9):121. doi: 10.3390/membranes9090121 (PMC6780848; doi:10.3390/membranes9090121)
Supplement: Supplementary file 1 [file membranes-09-00121-s001.pdf]

membranes

Supplementary Materials: Characterization of initial fouling layer on membrane surface in membrane bioreactor: Effects of permeation drag

Shengli Wang[1a], Xin Lu[1a], Lanhe Zhang[1], Jingbo Guo[2] and Haifeng Zhang[1,*]

Materials and Methods

Surface Thermodynamics Analysis

Surface tension components are calculated by the extended Young equation (Hoek and Agarwal, 2006):

$$(1+\cos\theta)\gamma_{l}^{Tot} = 2\left(\sqrt{\gamma_{s}^{LW}\gamma_{l}^{LW}} + \sqrt{\gamma_{s}^{+}\gamma_{l}^{-}} + \sqrt{\gamma_{l}^{+}\gamma_{s}^{-}}\right) \quad (1)$$

$$\gamma^{Tot}=\gamma^{LW}+\gamma^{AB}=\gamma^{LW}+2\sqrt{\gamma^{+}\gamma^{-}} \quad (2)$$

where θ is the contact angle, γ^{TOT} is the total surface tension, γ^{LW} is the Lifshitz-van der Waals component, γ^{AB} is the acid-based surface tension component, and γ^{+} and γ^{-} are the electron-acceptor and electron-donor components, respectively. The subscripts *s* and *l* represent the solid surface and the liquid, respectively.

With the equations above, the adhesion free energy (ΔG_{adh}^{Tot}) between initial fouling layer and membrane and the cohesion free energy (ΔG_{coh}^{Tot}) between foulants are evaluated with the following equations (Wang et al., 2013):

$$\Delta G_{d_0}^{LW} = -2\left(\sqrt{\gamma_{m/f}^{LW}} - \sqrt{\gamma_{w}^{LW}}\right)\left(\sqrt{\gamma_{f}^{LW}} - \sqrt{\gamma_{m/f}^{LW}}\right) \quad (3)$$

$$\Delta G_{d_0}^{AB} = 2\left[\sqrt{\gamma_{w}^{+}}\left(\sqrt{\gamma_{f}^{-}}+\sqrt{\gamma_{m/f}^{-}}-\sqrt{\gamma_{w}^{-}}\right)+\sqrt{\gamma_{w}^{-}}\left(\sqrt{\gamma_{f}^{+}}+\sqrt{\gamma_{m/f}^{+}}-\sqrt{\gamma_{w}^{+}}\right)-\sqrt{\gamma_{f}^{-}\gamma_{m/f}^{+}}-\sqrt{\gamma_{f}^{+}\gamma_{m/f}^{-}}\right] \quad (4)$$

$$\Delta G_{d_0}^{EL}=\frac{\varepsilon_0\varepsilon_r\kappa}{2}\left(\xi_f^2+\xi_{m/f}^2\right)\left[1-\coth(\kappa d_0)+\frac{2\xi_f\xi_{m/f}}{\xi_f^2+\xi_{m/f}^2}\operatorname{csch}(\kappa d_0)\right] \quad (5)$$

$$\kappa=\sqrt{\frac{1000N_A e^2}{\varepsilon_0\varepsilon_r K_B T}2I} \quad (6)$$

$$\Delta G_{d_0}^{tot} = \Delta G_{d_0}^{LW} + \Delta G_{d_0}^{AB} + \Delta G_{d_0}^{EL} \quad (7)$$

where d_0 is a minimum equilibrium cut-off distance (d_0 = 0.158 nm), $\varepsilon_0\varepsilon_r$ is the permittivity of the sludge suspension, ξ is the surface zeta potential of membrane (subscript *m*) and foulants (subscript *f*), κ is the inverse Debye screening length, N_A is the Avogadro number (6.0×10^{23} mol^{-1}) and I is the ionic strength (mol/L), ε_0 is the vacuum permittivity (8.85×10^{-12} $CV^{-1}m^{-1}$), ε_r is the

relative permittivity of the background solution (80 for water), e is the elementary charge (1.60×10^{-19} C), K_B is Boltzmann constant (1.38×10^{-23} J/K), T is the absolute temperature (K).

The free interaction energy (ΔG_{sws}) between two identical surfaces in water can be considered as an indicator of surface hydrophobicity/hydrophilicity. The ΔG_{sws} can be calculated as Equation (8) (Hong et al., 2014):

$$\Delta G_{sws} = -2\left(\sqrt{\gamma_s^{LW}} - \sqrt{\gamma_w^{LW}}\right)^2 - 4\left(\sqrt{\gamma_s^+\gamma_s^-} + \sqrt{\gamma_w^+\gamma_w^-} - \sqrt{\gamma_s^+\gamma_w^-} - \sqrt{\gamma_w^+\gamma_s^-}\right) \quad (8)$$

Extended DLVO Theory

The calculations of ΔG^{AB}, ΔG^{LW} and ΔG^{EL} between initial fouling layer and membrane surface at separation distance (d) are calculated as Equations (9)–(11) (Lin et al., 2014; Chen et al., 2015; Cai et al., 2016).

$$\Delta G^{LW}(d) = \Delta G_{d_0}^{LW} \frac{d_0^2}{d^2} \quad (9)$$

$$\Delta G^{AB}(d) = \Delta G_{d_0}^{AB} \exp(\frac{h_0 - h}{\lambda}) \quad (10)$$

$$\Delta G^{EL}(d) = \kappa \xi_m \xi_f \varepsilon_0 \varepsilon_r \left(\frac{\xi_f^2 + \xi_m^2}{2\xi_f \xi_m}(1 - \coth(\kappa d)) + \frac{1}{\sinh(\kappa d)} \right) \quad (11)$$

The calculations of U_{fwm}^{LW}, U_{fwm}^{AB}, U_{fwm}^{EL} and U_{fwm}^{Tot} between initial fouling layer and membrane surface at separation distance (d) are calculated as Equations (12)–(15) (Lin et al., 2014; Chen et al., 2015; Cai et al., 2016).

$$U_{fwm}^{LW}(D) = \int_0^{2\pi}\int_0^R \Delta G^{LW}(D + R + z - \sqrt{R^2 - r^2} - f(r,\theta))rdrd\theta \quad (12)$$

$$U_{fwm}^{AB}(D) = \int_0^{2\pi}\int_0^R \Delta G^{AB}(D + R + z - \sqrt{R^2 - r^2} - f(r,\theta))rdrd\theta \quad (13)$$

$$U_{fwm}^{EL}(D) = \int_0^{2\pi}\int_0^R \Delta G^{EL}(D + R + z - \sqrt{R^2 - r^2} - f(r,\theta))rdrd\theta \quad (14)$$

$$U_{fwm}^{XDLVO}(d) = U_{fwm}^{LW}(d) + U_{fwm}^{AB}(d) + U_{fwm}^{EL}(d) \quad (15)$$

$$f(r,\theta) = zsin(\pi r\cos\theta / 2z + \varphi) \quad (16)$$

where D is the closest distance between a particle and membrane surface; R is the particle radius; z is the roughness of membrane surface; r is the radius of differential circular ring on particle surface; $d\theta$ is the differential angle of the differential circular arc in the circular ring, φ is assumed to be zero for simplicity in this study.

The double integrals were estimated through composite Simpson's rule (Lin et al., 2014; Chen et al., 2015; Cai et al., 2016).

$$\begin{aligned} \int_a^b\int_c^d f(x,y)dxdy &= \sum_{i=1}^{m}\sum_{j=1}^{n}\int_{x_{2i-2}}^{x_{2i}}\int_{y_{2j-2}}^{y_{2j}} f(x,y) \\ &\approx \frac{hk}{9}\sum_{i=1}^{m}\sum_{j=1}^{n}(f_{2i-2,2j-2} + f_{2i,2j-2} + f_{2i,2j} + f_{2i-2,2j}) \\ &+ 4(f_{2i-1,2j-2} + f_{2i,2j-1} + f_{2i-1,2j} + f_{2i-2,2j-1}) + 16f_{2i-1,2j-1} \end{aligned} \quad (17)$$

where certain point $x_1 = a$, $x_i = x_1 + ih$ (i=1, 2,…, 2m+1) and $y_1 = b$, $y_i = y_1 + jk$ (j=1, 2,…, 2n+1) were used to subdivide the interval [a,b] of variable x and the interval [c,d] of variable y in a double integral,

respectively ($h = (b-a)/2m$), and $k = (d-c)/2n$), and m and n are the number of segments for the variable interval of x and y, respectively.

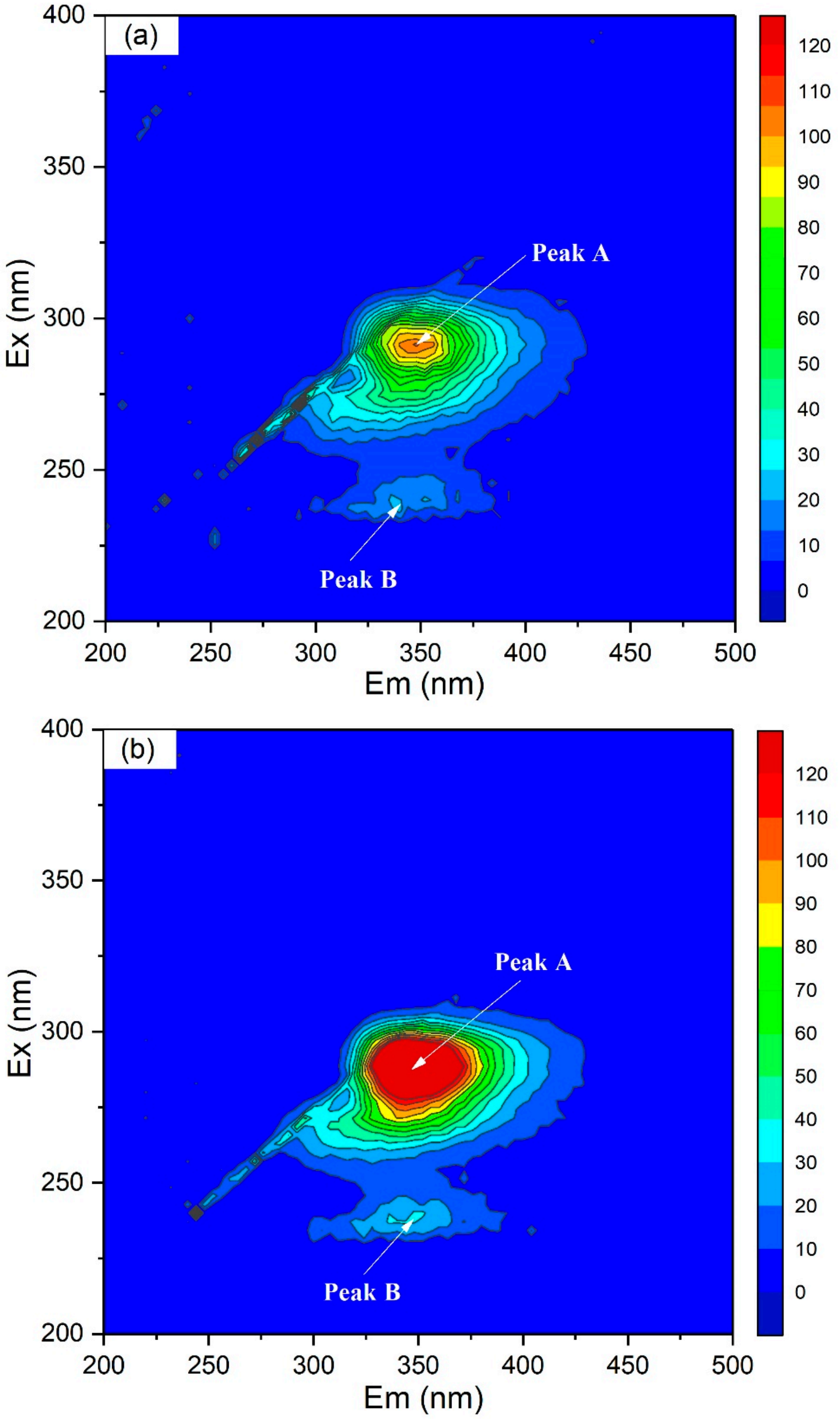

Figure S1. EEM fluoreacence spectra of EPS extracted from the initial fouling layer on membrane with no flux (0 L/m^2·h) and normal flux (10 L/m^2·h).

References

1. Chen J.; Mei R.; Shen L.; Ding L.; He Y.; Lin H.; Hong H. Quantitative assessment of interfacial interactions with rough membrane surface and its implications for membrane selection and fabrication in a MBR. *Bioresour. Technol.* **2015**, *179*, 367–372.
2. Cai H.; Fan H.; Zhao L.; Hong H.; Shen L.; He Y.; Lin H.; Chen J. Effects of surface charge on interfacial interactions related to membrane fouling in a submerged membrane bioreactor based on thermodynamic analysis. J. *Colloid Interface Sci.* **2016**, *465*, 33–41.

3. Hoek EMV.; Agarwal GK. Extended DLVO interactions between spherical particles and rough surfaces. *J. Colloid Interface Sci*. **2006**, *298*, 50–58.
4. Hong H.; Zhang M.; He Y.; Chen J.; Lin H. Fouling mechanisms of gel layer in a submerged membrane bioreactor. *Bioresour. Technol*. **2014**, *166*, 295–302.
5. Lin H.; Zhang M; Mei R.; Chen J.; Hong H. A novel approach for quantitative evaluation of the physicochemical interactions between rough membrane surface and sludge foulants in a submerged membrane bioreactor. *Bioresour. Technol.* **2014**, *171*, 247–252.
6. Wang Q.; Wang Z.; Zhu C.; Mei X.; Wu Z. Assessment of SMP fouling by foulant-membrane interaction energy analysis. *J. Membr. Sci.* **2013**, *446*, 154–163.

© 2019 by the authors. Submitted for possible open access publication under the terms and conditions of the Creative Commons Attribution (CC BY) license (http://creativecommons.org/licenses/by/4.0/).
